# Editorial: Updates on toll-like receptors in cancer immunity and immunotherapy

**DOI:** 10.3389/fimmu.2023.1331317

**Published:** 2023-11-13

**Authors:** Bettina Hoden, Yasuhiro Nagai, Laura Schuettpelz, Dekai Zhang

**Affiliations:** ^1^ Center for Infectious and Inflammatory Diseases, Institute of Biosciences and Technology, Texas A&M University, Houston, TX, United States; ^2^ Cancer Research Unit, Sumitomo Pharma Co. Ltd., Osaka, Japan; ^3^ Department of Pediatrics, Washington University School of Medicine, St. Louis, MO, United States

**Keywords:** toll-like receptor (TLR), cancer, cancer immunity, immunotherapy, TLR signaling

Toll-like Receptors (TLRs) are a class of Pattern Recognition Receptors (PRRs) capable of recognizing pathogen-associated molecular patterns (PAMPs) (1) and damage-associated molecular patterns (DAMPs) (2) to initiate immune responses. TLRs are crucial for the detection of infections, and activating downstream signaling pathways that lead to the production of pro-inflammatory cytokines and interferons, thereby initiating host defense mechanisms against a wide range of pathogens (3). Moreover, TLRs are involved in the crosstalk between the innate and adaptive immune systems, as their activation can influence the development of antigen-specific adaptive immune responses (4). TLR ligands differentially regulate the function of dendritic cells that play a central role during priming and activation of naive T cells (5). Understanding the complex interplay of TLRs in immune regulation not only contributes to our comprehension of host defense mechanisms but also has implications for the development of vaccines, immunotherapies, and treatments for various autoimmune and inflammatory disorders such as cancer.

Recent developments in the field of TLRs and cancer immunity underscore the dual role of TLRs in cancer development: promoting or inhibiting tumor growth. TLR agonists are being explored as adjuvants in cancer vaccines and immunotherapies, thereby enhancing the activation of dendritic cells and adaptive immune responses against cancer. However, the intricate interplay between TLRs and the tumor microenvironment has been increasingly recognized, with some TLRs implicated in supporting an immunosuppressive milieu that benefits tumor growth. Velasco et al. demonstrated that TLR signaling blockade significantly decrease COPD-like inflammation dependent tumor onset in the lung using k-ras driven lung adeno-carcinoma model. Researchers are now delving into the specific TLR subtypes, their differential effects on immune cells, and the development of TLR-targeted therapies that could either enhance the immune response against cancer or attenuate immunosuppressive signals, all of which hold significant promise in advancing cancer immunotherapy strategies (Hoden et al.). Interestingly, Liu et al. showed that LPS signaling enhanced tumor apoptotic activity of IAP targeting therapy, suggesting TLR signaling may use “synthetic lethal” approach for reducing its toxicity.

In recent years much scientific interest has focused on improving the efficacy of cancer immunotherapy. In the realm of cancer immunotherapy, TLRs are garnering increased attention as potential therapeutic targets. TLR agonists are being explored for their capacity to stimulate innate immune responses, such as the activation of dendritic cells and natural killer cells, which can bolster antitumor immunity. Combining TLR agonists with other immunotherapies like checkpoint inhibitors or adoptive cell therapies holds promise in creating synergistic effects to enhance cancer treatment outcomes (Hoden et al.). However, there is ongoing research to better understand the nuances of TLR signaling in the context of different cancer types and patient populations to optimize their use in personalized cancer immunotherapies. Furthermore, efforts to mitigate potential side effects and enhance the specificity of TLR-targeted treatments are also underway, indicating a dynamic and evolving field within cancer immunotherapy. Ota et al. tried to achieve efficacy- safety margin by utilizing small molecule TLR7 specific agonist which shows rapid clearance from the body.

The therapeutic prospects for TLRs are exceptionally promising, as they represent a critical avenue in both the understanding and application of immunotherapy. In this topic there are 3 review articles discussing the strategy of clinical application by targeting TLR signaling, especially in the oncology field. Hoden et al. focuses on TLR agonists for use in lung cancer. More broadly, Yang et al. focuses more on the mechanisms and cutting-edge technologies of various tumor types and Mukheerjee et al. explores cancer type specific strategies. Ongoing research is likely to uncover novel TLR subtypes and ligands, shedding light on their nuanced roles in diverse disease contexts, including cancer, infectious diseases, and autoimmune disorders. Additionally, the development of more refined TLR-targeted therapies, such as selective agonists or antagonists, offers the potential for precise modulation of immune responses, minimizing unwanted side effects. Harnessing TLRs as adjuvants in combination with emerging immunotherapies and personalized medicine approaches may further revolutionize the treatment of various conditions, ultimately paving the way for more effective and tailored therapeutic interventions. This evolving field holds great promise for the continued advancement of immunotherapy and precision medicine in the years to come.

## Author contributions

BH: Writing – original draft. YN: Writing – review & editing. LS: Writing – review & editing. DZ: Writing – original draft, Writing – review & editing.
